# Fruit Availability and Maternal Energy Expenditure Associated With Infant Independence in an Arboreal Primate (*Colobus angolensis ruwenzorii*)

**DOI:** 10.1002/ajp.70056

**Published:** 2025-06-12

**Authors:** Samantha M. Stead, Edward Mujjuzi, Julie A. Teichroeb

**Affiliations:** ^1^ Department of Anthropology University of Toronto Scarborough Toronto Ontario Canada; ^2^ Rwenzori Colobus Project Nabugabo Masaka District Uganda

**Keywords:** allomaternal care, food availability, infant development, infant handling, multi‐level society

## Abstract

A range of ecological and social factors have been shown to affect early‐life behaviour in mammals. Primate infants are altricial and thus unable to move independently at birth. As a result, infants in some species are continuously held or carried (handled) by their mother or another caregiver (allomother). Variation in the amount of time infants move independently can provide insight into the costs and benefits associated with this developmental milestone. In this study, we sought to investigate what environmental conditions are associated with independence in an arboreal primate, the Rwenzori Angolan colobus (*Colobus angolensis ruwenzorii*). We followed 29 infants from birth until 4 months, collecting data on whether the infant was handled or independent. We report the age‐sex make up of infant handlers and show that fruit availability was positively associated with infant independence, and maternal movement frequency was negatively associated with infant independence. We suggest that greater maternal energy balance during early infancy allows mothers to divert more energy to infants, promoting their independent movement. Further research should assess the maternal physiology underlying these trends and whether earlier independent movement has long‐term fitness effects.

## Summary

- Primates must learn to locomote independently during early life.
- We followed Angolan colobus from birth until 4 months.
- Fruit availability was positively associated with infant independence.
- Maternal energy expenditure was negatively associated with infant independence.

## Graphical abstract

Infant being handled by mother.

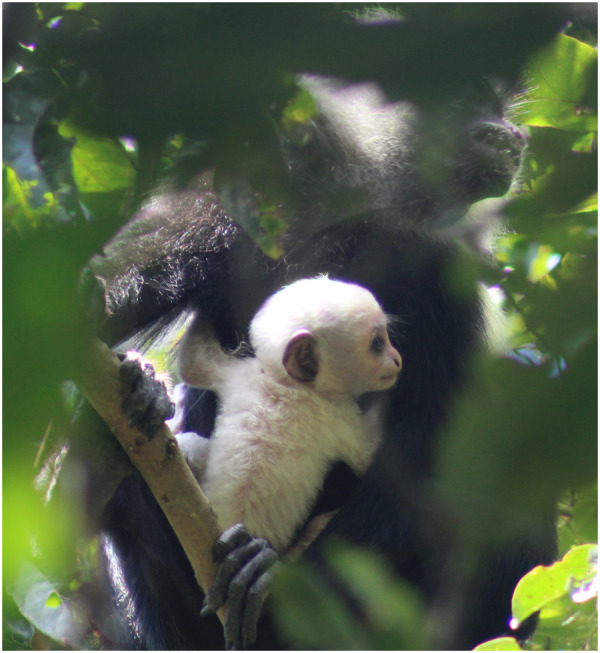

## 1 Introduction

Ecological and social factors can affect early‐life behaviour in mammals (Graham and Burghardt 2010; Hinde 2013; Bezanson 2017). For example, snowshoe hare (*Lepus americanus*) juveniles born into more predator‐dense environments tend to be less active and spend more time under cover (Sheriff et al. 2010; Lavergne et al. 2019) and chimpanzee (*Pan troglodytes*) juveniles living in habitats with greater fruit abundance tend to spend more time playing than when fruit is less abundant (Moebius et al. 2019). Early‐life behaviours can impact growth and development, and investigations into how environmental factors affect these behaviours can uncover the trade‐offs underlying these processes (McNamara and Houston 1996; Stearns 1992).

Primate neonates are altricial and unable to support their own weight at birth (Grand 1992). In many nonhuman species, infants must cling to their mother or another caregiver (hereafter “allomother”) while they forage, travel, rest, and socialize (Ross 2001). This is particularly important in arboreal primates, as unsupported neonates are at risk of fatal or permanently debilitating falls. Clinging infants' immediate environments are controlled by their caregiver(s). Once infants learn to move independently, they can begin to explore their physical and social environment, making this an important developmental milestone. Until then, independent movement consists of short forays within reach of the caregiver, allowing the infant to explore in relative safety while its musculoskeletal system develops and its motor control improves (Young and Shapiro 2018, Figure 1).

**Figure 1:**
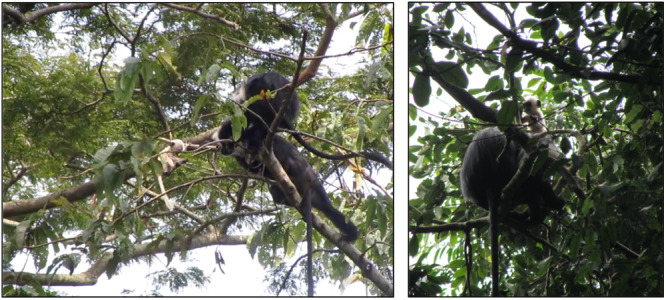
Left and right photos of infants under 4 months of age moving independently near a caregiver (media by Samantha Stead).

Several researchers have documented variation in the age at which infants were first observed to be independent of their mother (e.g., *Ateles geoffroyi*, Arbaiza‐Bayona et al. 2022; *Alouatta palliata*, Lyall 1996; *Trachypithecus leucocephalus*, Zhao et al. 2008; *Pan troglodytes*: Doran 1992; *Gorilla gorilla*, Doran 1997) and/or the age at which they began to move independently for more than 80% of their daily movement (review: Young and Shapiro 2018). As well, many researchers have assessed variation in maternal‐infant proximity (e.g., *Propithecus coquereli*, Ross and Lehman 2016; *Ateles geoffroyi*, Arbaiza‐Bayona et al. 2022; *Macaca mulatta*, Hinde and Atkinson 1970; *M. fuscata*, Schino et al. 1993; *Cercopithecus mitis*, Förster and Cords 2004; *Pongo pygmaeus*, Mendonça et al. 2017). However, very few studies have looked specifically at variation in being held or carried (hereafter “handled”) versus independent during early life (though see Altmann and Samuels 1992). Understanding such variation may provide insight into how ecological and social conditions shape the early‐life behaviours of primate infants.

Infant independence is accompanied by costs and benefits in arboreal primates. There are safety risks associated with independence, such as falling and being more easily targeted by predators and infanticidal males (Chalmers 1972; Young and Shapiro 2018). Additionally, independence is undoubtedly more energetically costly for an arboreal infant than being handled, as infants must engage their muscles to support their own weight and maintain balance on thin, flexible branches. Independent infants are also unlikely to be stationary and there is evidence that locomotor play is energetically costly (Miller and Byers 1991; Pellegrini et al. 1998). On the other hand, locomotor play likely has benefits for the infant, such as accelerated motor skill acquisition (Byers and Walker 1995; Bekoff 1988; Burghardt 2014; Berghänel et al. 2015), which can improve flight/fight abilities and potentially increase dominance and survival in some species (Nunes et al. 2004; Cameron et al. 2008; Fagen and Fagen 2009; Blumstein et al. 2013). Thus, despite the associated costs, there are likely benefits to independent movement during early life.

Before weaning, infants rely entirely on their mother's milk for energy and thus maternal energy balance (net difference between caloric intake and expenditure, Emery‐Thompson 2017) will dictate the resources available to infants (Power and Schulkin 2016). Indeed, several primate studies found that nutritionally constrained mothers had infants that were less active (e.g., Rosenblum and Paully 1984; Rahmanifar et al. 1993). Infant handling is energetically costly for handlers due to both the increased energetic costs of movement as well as a decrease in feeding efficiency (Goldizen 1987; Tardif 1996; Achenbach and Snowdon 2002; Lappan 2009). Allomothers can relieve mothers of handling duties and theoretically improve the mothers' energy balance. However, no primate studies have investigated the impact of allomaternal care on maternal energy balance using physiological measures (e.g., urinary c‐peptides, fecal glucocorticoid metabolites, Emery‐Thompson 2017).

In this study, we focused on Rwenzori Angolan colobus at Nabugabo, a population where individuals spend virtually all of their time in trees. Our aims were to (1) document changes in infant handling frequency throughout the first 4 months of life; (2) document variation in allomaternal handler identity; both male and female subadult and adult allomothers frequently handle infants in this population (S.M.S., E.M. personal observations), but proportions of handling by each of these categories is unknown; and (3) investigate how ecological and social factors impact infant independence during the first 4 months of life.

Rwenzori Angolan colobus monkeys are unique among African colobines due to their formation of multi‐level societies, which are complex social systems with multiple tiers of nonrandom association (Stead and Teichroeb 2019; Miller et al. 2020). Core units, the most basic social unit, can range in size from four to 23 individuals and have one to eight reproductively active males (Stead and Teichroeb 2019). Though core units are regularly found in proximity to one another, they are socially and spatially distinct (Stead and Teichroeb 2019). Virtually all infant interactions occur with individuals living in the same core unit. Core units associate preferentially with other core units from the same clan (Adams et al. 2021). Clans from the same band share a home range (Teichroeb et al. 2022). Thus, at Nabugabo, there are at least three tiers of nonrandom association: the core unit, the clan, and the band (Stead and Teichroeb 2019). Survival analysis found that when a male(s) immigrated from another core unit in the band to an infant's core unit, there was an increased likelihood of that infant dying (Stead et al. 2025). The authors suggest that males occasionally target and kill unrelated infants in this population as a reproductive strategy to induce estrous in the mother and sire their own offspring (i.e., sexual selection hypothesis, Blaffer Hrdy 1974), but that it is uncommon. However, this remains speculative and to date there have been no direct observations of infanticide by males at Nabugabo or in any other populations of Rwenzori Angolan colobus. In terms of diet, the colobus at Nabugabo feed primarily on young leaves (65%), but fruits (31%) are the only food that is positively selected for when available (Arseneau‐Robar et al. 2021). Large terrestrial predators have been extirpated at Nabugabo, but snakes (e.g., *Dendroaspis polylepis*, *Dispholidus typus, Python sebae*) and crowned hawk eagles (*Stephanoaetus coronatus*) still pose a threat, as do local people's dogs (*Canis lupus familiaris*) (Mitani et al. 2001; Adams and Teichroeb 2020).

## 2 Hypotheses and Predictions

Our first hypothesis (H1) was that infants whose mothers had a lower energy balance would be less active, as there would be less energy available to fuel infant movement. We predicted that (1) infants would be less likely to be independent during periods of lower fruit availability (lower maternal energy intake), (2) infants whose mothers spent a greater proportion of scans moving (greater maternal energy expenditure) would be less likely to be independent, and (3) infants whose mothers received more allomaternal assistance during scans (less maternal energy expenditure) would be more likely to be independent.

Our second hypothesis (H2) was that infants living in riskier environments would be less likely to be independent, as independent infants are more vulnerable than handled ones. We predicted that (1) infants living in core units with immigrant males would be less likely to be independent, as research on this population and a closely related species indicates that infanticide by males could pose a threat (*Colobus vellerosus*: Sicotte and Teichroeb 2008; *C. angolensis ruwenzorii*: Stead et al. 2025), (2) infants would be less likely to be independent when their core unit was in association with more core units due to the proximity of unfamiliar (potentially infanticidal) males, and (3) infants living in smaller core units would be less likely to be independent, as predation risk is greater in smaller units because there are fewer individuals to detect predators and to dilute predation risk (van Schaik 1983; Hamilton 1971).

## 3 Methodology

### 3.1 Ethical Note

All research reported in this manuscript adhered to the legal requirements of Uganda and received clearance from the Uganda Wildlife Authority (Permit: UWA/COD/96/02) and the Uganda National Council of Science and Technology (Permit: NS537). This study also received clearance from the University of Toronto Animal Care Committee (Protocol: 20011416) and adhered to the American Society of Primatologists' Principles for the Ethical Treatment of Nonhuman Primates.

### 3.2 Study Site and Population

Our study population inhabits a forest fragment near Lake Nabugabo in Central Uganda, Masaka District (0° 20' S and 31° 52 E). The monkeys occupy patches of primary and secondary moist evergreen forest with relatively flat terrain (elevation range: 1,134–1,167 m a.s.l.). Wet and dry seasons occur biannually in the Masaka District. During the study period, there was a mean annual rainfall of 1217 mm (953–1477 mm) and a mean annual temperature of 21.9°C (21.6°C–22.2°C) (data from Masaka, 12.5 km away, https://www.worldweatheronline.com). An earlier study showed that the 95% home range size estimate of the band was 1.75 km^2^ and the mean daily path length of core units was 386.67 m (range: 44.0–1096.5 m) (Teichroeb et al. 2022). Based on data from a closely related species (*C. vellerosus*), gestation is approximately 26 weeks (Vayro et al. 2016) and lactation can range from 39 to 67 weeks (MacDonald 2011; Crotty 2016). Reproduction is nonseasonal in this population (SMS unpublished data).

### 3.3 Data Collection

From September 2019 to April 2021 (18 months), we followed 29 mother‐infant dyads living in 10 different core units. We followed each infant from birth (or whenever it was first found) until it reached 4 months of age (120 days), went missing, or the study period ended. We focused on infants that were ≤ 4 months of age because data from a closely related species (*C. vellerosus*) indicated that they spend negligible time (0%–1%) consuming solid foods (MacDonald 2011). Throughout the study period, we attempted to locate each core unit of our study band on a weekly basis to note any new births, as well as any changes in core unit composition. To estimate birth dates, we noted the date that the core unit was last observed without an infant and the date that the infant was first observed. We then chose the date in between these two as the birth date. Birth date error was determined by calculating the number of days between when the infant was first observed and when the unit was last observed without the infant. This number was then divided by 2. Birth date error was on average 8 days (range: 0–27 days). We attempted to rotate between each of the study core units daily to evenly distribute our data across mother‐infant dyads; however, this was not always possible, as some core units were more difficult to locate than others. Infant sex was determined by observing their genitalia; infants with a penis were noted as male and those without a penis were noted as female. We were unable to assign some infants to a sex due to poor visibility.

We collected behavioural scan data (*n* = 3717, infant average = 128 ± 59 scans) on each of our study infants and their mothers from 08:00 (or whenever we located our target core unit) to 16:00. Every 15 min, we scanned the focal core unit for each of the study infants and their mothers (Altmann 1974). During these scans, we noted whether each study infant was being handled or independent. We defined handling as when an infant was being physically supported by a mature individual's legs and/or arms; an infant that was solely in body contact with an immature individual was not considered to be handled (Bales et al. 2000; Bădescu et al. 2015). Rwenzori Angolan colobus monkeys do not carry their infants dorsally, only ventrally. Focal data on infants (< 6 months old) in this population found that 68.0% of infant handling bouts were terminated by the infant moving out of the handler's lap, 31.8% were terminated by another handler taking the infant, and only 0.22% were terminated by the handler (*n* = 444 infant handling bouts) (SMS unpublished data, see Stead et al. 2021 for methods). We are thus confident that infants that were independent during a scan had chosen to move out of a handler's lap, representing an active infant. If the infant was being handled, we noted the handler's identity. At each 15‐min scan, we also noted whether the core unit was stationary or moving. Every 2 h, we noted which core unit(s) was in association (within 50 m of) with our target core unit to capture the broader social environment of our study infants and their mothers.

#### 3.3.1 Food Availability

To assess food availability, we first determined the tree species composition of the forest fragment occupied by our study band. We used 17 line transects cut at 100 m intervals across the forest fragment. Transects varied in length (range: 29–671 m) and were cut either until the edge of the fragment was reached or we moved into the swamp on the north‐west side of the forest and standing water became more than 30 cm deep (Teichroeb et al. 2019). We identified and measured all trees ≥ 10 cm diameter at breast height (DBH) within 5 m on each side of the transects. For trees with multiple stems ≥ 10 cm DBH, we measured all stems and consolidated them by taking the square root of the sum of all squared DBHs (Nature Conservation 2006). The basal area covered by each tree species was calculated by summing the area covered by each sampled tree (A = πr^2^) and dividing by the number of hectares sampled.

The availability of unripe and ripe fruit was recorded monthly throughout the study period. We monitored a random sample of at least three individual trees of each of the 20 tree species that the colobus feed on (*n* = 66 individual trees, Arseneau‐Robar et al. 2021). We indexed the approximate abundance of fruits by noting the percent of the canopy comprised of fruit using a scale from 0 to 4, where 0 = the plant part is not present, 1 = 1–25%, 2 = 26–50%, 3 = 51–75%, and 4 = 76–100% (Sun et al. 1996). The numbers recorded also considered the proportion of the canopy covered in that plant part. For example, for a tree where half the fruit present was ripe and half was unripe, and where the crown was only half covered in fruit, we gave a phenology score of 1 for ripe fruit and 1 for unripe fruit (total fruit score = 2, indicating that 50% of the tree was covered; for details see Teichroeb et al. 2021: 215). For each tree species, phenology scores were averaged among all the trees sampled each month. We then calculated a monthly fruit availability index (FAI) using the formula, FAI = average phenology score for species i × basal area for species i, finally summing the scores for all tree species to obtain the monthly value (Dasilva 1994; Fashing 2001).

### 3.4 Data Analyses

To determine what ecological and social factors affected the likelihood that an infant was independent, we ran a generalized linear mixed model (GLMM) with a binomial distribution and logit link function. The response variable was whether the infant was handled or not. We include the following predictor variables in the full model: infant age, core unit activity (moving vs. stationary), infant sex, monthly fruit availability, monthly allomaternal assistance (total # scans handled by allomother/total # scans handled), monthly maternal energy expenditure (total # scans moving/total number of scans), number of core units in association, immigrant males (Y/N), and core unit size. We included a random effect for mother‐infant dyad and core unit to account for repeated measures of maternal‐infant dyads living in the same core units. We excluded UNI4, UNI6, and UNI7 from this analysis, as we were only able to observe them for 1 day each before they died. As well, due to missing monthly fruit availability data, monthly maternal activity budget data, and/or weekly core unit composition data, we had to exclude some scans from our data set. This left us with 2,394 scans (mother‐infant dyads = 23) for our model.

GLMMs were fit using the ‘glmer’ function from the lme4 package, with maximum likelihood estimation and the Laplace approximation (Bates et al. 2015). To identify the model that best explained the relationship between predictors and response variables, we used the ‘dredge’ function from the MuMIn package (Bartoń 2024) to generate all subsets of the global GLMM. We then selected the top models (ΔAIC ≤ 2) and performed a model averaging procedure using the ‘model. avg’ function (Table 1). We performed a bootstrap analysis to confirm the reliability of the fixed effect estimates from the GLMMs (1000 iterations). We obtained confidence intervals using the ‘bootMer’ function of the package lme4. We confirmed linear relationships between continuous predictors and the log‐odds of infant independence by using the ‘simulateResiduals’ function in the DHARMa package to generate residuals and then plotting them against predictors (Hartig 2024). Additionally, tests for overdispersion and zero inflation were conducted using ‘testDispersion’ and ‘testZeroInflation’ functions, respectively. We calculated the variance inflation factors (VIF) for the top models using the ‘vif’ function from the ‘car’ package (Fox and Weisberg 2019). In our models, all predictors had VIF values below 2 (1.58), indicating no concerns with collinearity. Lastly, we examined a QQ‐plot and histogram of the random effects residuals to confirm that the assumption of normality was met. All analyses were done in R (R Core Team 2024).

## 4 Results

**Figure 2:**
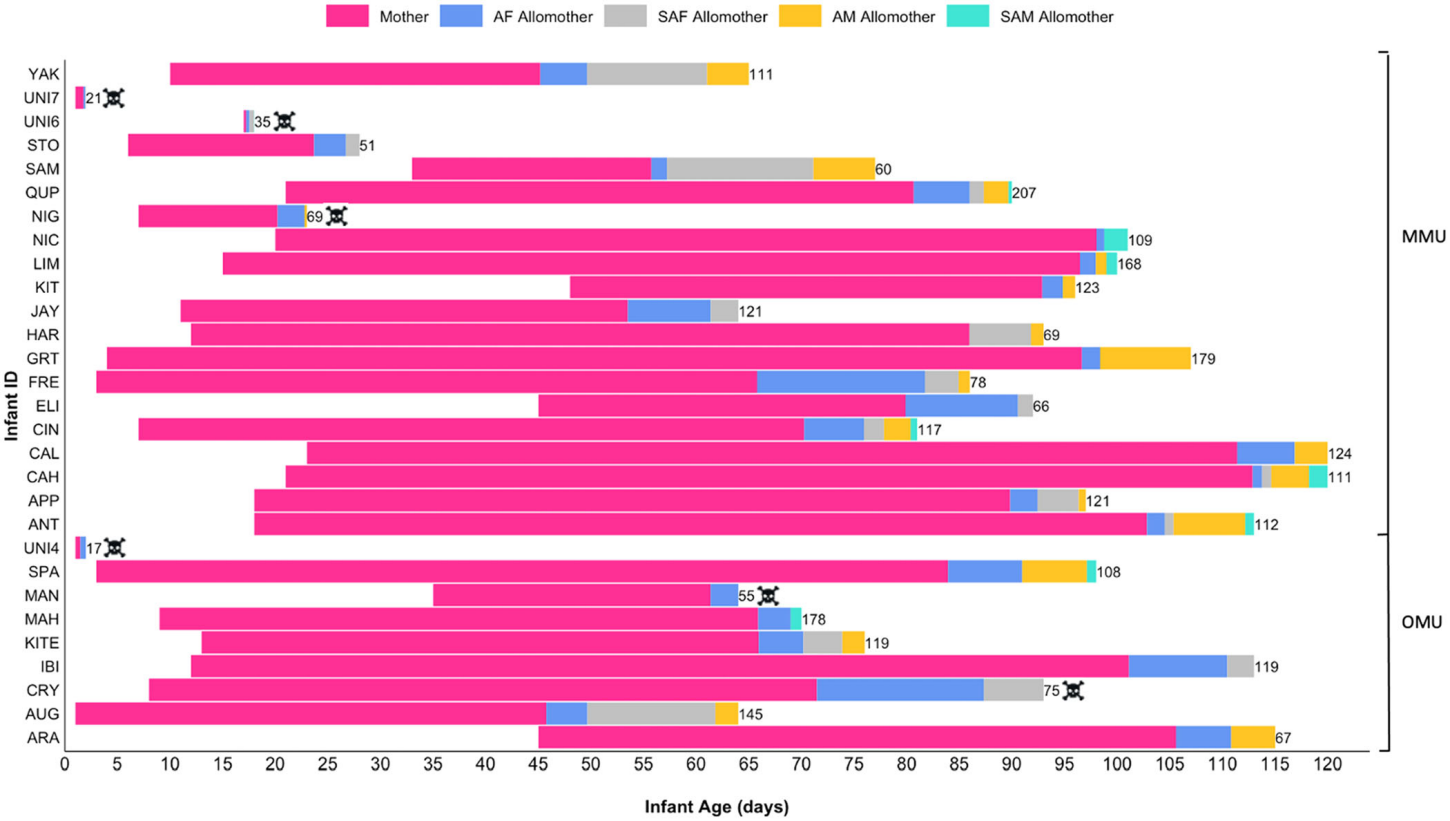
Bar graph depicting variation in handling by allomothers for each of the 29 study infants. Each bar is displayed across the ages (x‐axis) during which data were collected for each infant (y‐axis). Numbers to the right of each bar indicate the number of scans that each infant was handled by a known allomother. Colours are proportional to the number of scans that each infant was handled by its mother (pink), an adult female (AF) allomother (blue), a subadult female (SAF) allomother (grey), an adult male (AM) allomother (orange), and a subadult male (SAM) allomother (turquoise). The skull and crossbones indicate infants that died before 4 months of age. The upper 20 infants were in multi‐male units (MMU) and the lower nine infants were in one‐male units (OMU).

We collected scan data on 29 mother‐infant dyads (7 females, 19 males, three unknown) living in 10 different core units that ranged in size from six to 19 monkeys (1–6 adult males and 2–5 adult females). Out of the 29 infants, six died before reaching 120 days, all of unknown causes (Figure 2). Out of the scans where the handler was identified (*n* = 2935, range of scans per infant: 17–207, Figure 2), infants were handled by allomothers in 15.6% (proportion of scans per infant: 3.7%–77.1%): the handler was an adult female in 7.5% of the scans (range per infant: 0%–52.9%), a subadult female in 4.6% (range per infant: 0%–42.9%), an adult male in 2.9% (range per infant: 0%–13.3%), and a subadult male in 0.5% (range per infant: 0%–2.9%) (Figure 2). Each infant was handled by an average of four different allomothers (range per infant: 2–6). Five of the 20 infants living in multi‐male core units were handled by two different adult males, one infant by three. Two of the infants were handled by male(s) that immigrated into their core unit after they were conceived.

### 4.1 Infant Independence

**Figure 3:**
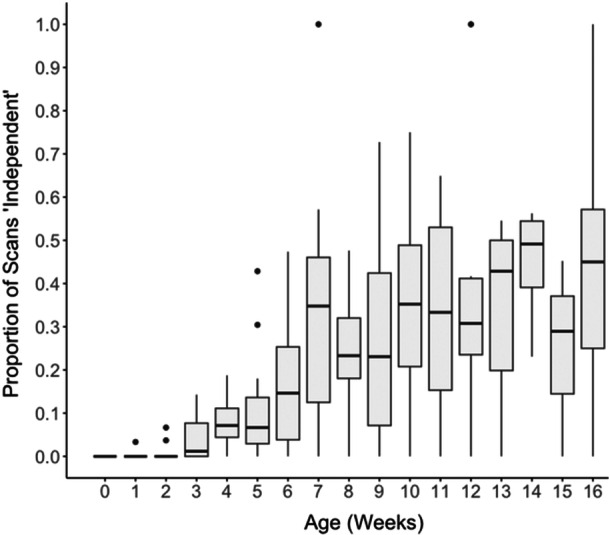
Bar plot depicting the proportion of weekly scans that an infant was independent. Each box represents the interquartile range (IQR) for infants, with the median represented by the horizontal line within the box. Whiskers extend to the smallest and largest values within 1.5 times the IQR. Data points beyond the whiskers represent outliers.

Infants were visible in 3717 scans; they were handled in 2985 (80.3%) and independent in 732 (19.7%). The proportion of independent scans per week had an overall increasing trend with age, with variability between infants (Figure 3). The youngest age at which an infant was observed to be independent was 11 days.

The model‐averaged results indicate that several factors were significantly associated with the likelihood of infant independence (Tables 1 and 2). As expected, infant age was positively associated with likelihood of infant independence (*β* = 1.35, 95% CI = [1.09, 1.61]). As well, infants were more likely to be independent when their core unit was stationary versus moving (*β* = 1.47, 95% CI = [1.05, 1.90]). Monthly fruit availability was positively associated with independence, indicating that infants were more likely to be independent in months when fruit was more abundant (*β* = 0.40, 95% CI = [0.14, 0.67]). Monthly maternal movement was negatively associated with infant independence, indicating that infants were more likely to be handled during months when mothers spent more scans moving (*β* = −0.28, 95% CI = [−0.51, −0.05]). The following predictors were not significantly associated with infant independence: infant sex (*β* = −0.58, 95% CI = [−1.99, 0.83]), monthly allomaternal care (*β* = −0.16, 95% CI = [−0.52, 0.20]), core unit size (*β* = −0.02, 95% CI = [−0.17, 0.12]), and the number of associated units (*β* = −0.002, 95% CI = [−0.05, 0.04]) The bootstrap model (1000 iterations) confirmed these results.

**Table 1:** Coefficients for the top models (ΔAIC ≤ 2) included in the model averaging procedure.

Model	Infant age	Core unit activity (Stationary)	Fruit availability	Monthly maternal movement	Monthly allomaternal care	Immigrant males (Y)	Associated core unit #	Unit size	Infant sex (Male)	df	AICc	Weight
1	1.34	+	0.42	−0.31	−0.28				+	9	1873.0	0.13
2	1.32	+	0.40	−0.30	−0.27	+			+	10	1873.3	0.11
3	1.33	+	0.42	−0.30	−0.24					8	1873.4	0.11
4	1.39	+	0.39	−0.23						7	1873.5	0.10
5	1.40	+	0.39	−0.23					+	8	1873.9	0.08
6	1.38	+	0.37	−0.21		+			+	9	1874.1	0.07
7	1.37	+	0.38	−0.22		+				8	1874.5	0.06
8	1.35	+	0.43	−0.32	−0.27			−0.08	+	10	1874.6	0.06
9	1.40	+	0.40	−0.23				−0.12		8	1874.6	0.06
10	1.32	+	0.40	−0.29	−0.23	+				9	1874.7	0.06
11	1.35	+	0.43	−0.31	−0.23			−0.12		9	1874.7	0.06
12	1.33	+	0.41	−0.31	−0.29		−0.05		+	10	1874.8	0.05
13	1.33	+	0.41	−0.30	−0.27	+		−0.07	+	11	1874.9	0.05

*Note:* Global model: glmer(Independence~infant age + core unit activity + fruit availability + monthly allomaternal care + monthly maternal movement + immigrant males + associated core unit # + unit size + infant sex + (1|core unit/mother‐infant dyad).

**Table 2:** Model‐averaged coefficients for the full and conditional model.

Tested hypotheses	Predictor	Estimate (full)	Error (full)	*z*‐value (full)	Estimate (cond.)	Error (cond.)	*z*‐value (cond.)
Control	Infant age	1.35	0.13	10.22	1.35	0.13	10.22
	Core unit activity (Stationary)	1.47	0.22	6.83	1.47	0.22	6.83
	Infant sex (Male)	−0.58	0.72	0.81	−1.04	0.67	1.54
Maternal energy balance (H2)	Fruit availability	0.40	0.14	2.95	0.40	0.14	2.95
	Monthly maternal movement	−0.28	0.12	2.35	−0.28	0.12	2.35
	Monthly allomaternal care	−0.16	0.18	0.88	−0.26	0.17	1.54
Mortality risk (H3)	Associated core unit #	−0.002	0.02	0.11	−0.05	0.09	0.51
	Male immigration (Y/N)	0.35	0.68	0.51	0.99	0.83	1.19
	Unit size	−0.02	0.07	0.30	−0.10	0.13	0.75

## 5 Discussion

### 5.1 Maternal Energy Balance

Our hypothesis that infants whose mothers had a lower energy balance would be less active (H1) was supported. Infant independence was positively associated with monthly fruit availability and negatively associated with monthly maternal movement. Previous research in this population has found that most infant handling bouts are terminated by the infant moving out of the handler's lap or being taken by another handler (see methods). We are thus confident that infants that were independent during a scan had chosen to move out of a handler's lap, representing an active infant. Although we don't have behavioural data on what the infants were doing while moving independently, solitary play is a strong possibility. Several experimental and observational studies show that young mammals played more when food availability was greater (e.g., *Odocoileus virginianus*, Muller‐Schwarze et al. 1982; *Spermophilus beldingi*, Nunes et al. 1999; *Suricata suricatta*, Sharpe et al. 2002; *Saimiri sciurea*, Baldwin and Baldwin 1976; Stone 2008; *Macaca assamensis*, Berghänel et al. 2015; *Trachypithecus leucocephalus*, Li and Rogers 2004; *Theropithecus gelada*, Barrett et al. 1992; *Gorilla gorilla*, Richardson et al. 2025; *Pan troglodytes*, Moebius et al. 2019; *Homo sapiens*, Bock and Johnson 2004). We thus suggest that greater fruit availability and less maternal movement allowed more energy to be allocated to infant activity, leading to a greater probability of infant independence during these periods. During the first 4 months of life, colobus infants rely predominantly on their mother's milk for energy, and thus any increased energy availability would be via the mother. Hinde and Capitanio (2010) were the first to measure available milk energy (AME) in rhesus macaque (*M. mulatta*) mothers and link higher AME to more exploratory infants. They suggest that infants whose mothers had low available milk energy had a more cautious behavioural phenotype that minimized energy expenditure (Hinde and Capitanio 2010; Hinde 2013). Monthly allomaternal care frequency was not associated with an increased likelihood of independence during that month. In fact, the relationship was negative in all the top models in which this predictor was retained (Table 1). This result suggests that either (1) any energy that mothers saved due to allomaternal handling was not diverted to infant activity or (2) allomaternal handling does not improve maternal energy balance. Lastly, we note that our finding that monthly maternal movement was negatively associated with infant independence may not be a result of lower maternal energy balance. Instead, it is possible that moving mothers are more likely to be handling their infants so that they are not left behing compared to resting and feeding mothers, who may be more permissive of infant exploratory forays (note that feeding in colobus monkeys is usually done from a sitting position and they rarely move while foraging).

### 5.2 Mortality Risk

Our hypothesis that infants living in riskier environments would be less likely to be independent was not supported. Male immigration was not significant in our averaged model and was actually positively associated with likelihood of independence in the top models in which this predictor was retained (Table 1). We observed two infants being handled by adult males that immigrated into their core unit after they were conceived (*n* = 10 scans). These handling events were recorded during the first 2 weeks of the infants' lives. While these males were handling the infants, both infants and mothers appeared relaxed, suggesting that the males were not perceived as an infanticide threat. Research on golden snub‐nosed monkeys (*Rhinopithecus roxellana*) living in a multi‐level society found that 57.1% of infants were sired by extra‐unit males (Qi et al. 2020). It is possible that Rwenzori Angolan colobus females also mate outside of their core units to create confusion about their infants' genetic fathers, reducing the likelihood that their infants will be targeted by immigrant males. Indeed, extra‐unit copulations have been observed in the Nabugabo population (E.M., personal observation). The two immigrant males may have previously mated with the infants' mothers and believed (correctly or not) that they sired the infants that they handled. As well, relatedness between adult males in our study band is still unknown, but males tend to disperse between core units in the same band, and so it is likely that certain males are closely related, which would further deter infanticide. Thus, risk associated with immigration of males is likely dependent on a variety of factors, such as the mother's mating history as well as relatedness between adult males.

Association of the focal core unit with other core unit(s) (within 50 m) did not impact the likelihood that an infant was independent during a scan. Previous work found that core units associate non‐randomly with one another (Stead and Teichroeb 2019; Teichroeb et al. 2024), and so it is possible that core units choose to associate more with units that they perceive as non‐threatening (i.e., no potentially infanticidal males). It is also possible that 50 m was not close enough for infanticidal male(s) to pose a threat to infants and their mothers (Stead and Teichroeb 2019).

Lastly, our prediction that infants living in smaller core units would be less likely to be independent was not supported. Predation risk may not be high enough in this population to impact infant independence, as many terrestrial predators have been extirpated. It is also possible that smaller core units associate with other units when they anticipate higher predation risks (Adams and Teichroeb 2020), one of the proposed benefits of a multi‐level societies (Whitehead et al. 2012; Schreier and Swedell 2012). In summary, none of the proxies for mortality risk that we selected were associated with likelihood of infant independence.

### 5.3 Future Directions

Future research should investigate the maternal physiology underlying the reported trends (Hinde 2013; Sheriff and Love 2013; Stead et al. 2022). A study on rhesus macaques (*Macaca mulatta*) found that higher milk glucocorticoid levels were associated with more behaviourally cautious offspring (Hinde et al. 2015). Glucocorticoids are steroid hormones that are secreted in high amounts in response to stressors (McEwen et al. 1988; Sapolsky et al. 2000). Stressors are any stimulus that threatens (or is perceived to threaten) an animal's homeostasis, the physiological processes that maintain a steady state (McEwen and Wingfield 2003; Boonstra 2013). Hinde et al. (2015) suggest that milk glucocorticoids are a way for mothers with fewer energetic resources to signal to their infants that they should reduce their activity and prioritize growth. Circulating glucocorticoid levels and energy balance can be assessed non‐invasively by measuring fecal glucocorticoid metabolite levels and urinary C‐peptide levels, respectively (Sheriff et al. 2011; Emery‐Thompson 2017). Thus, it would be interesting to investigate (1) how fruit availability and maternal movement frequency affect nursing mothers' energy balance and glucocorticoid levels and (2) how nursing mothers' energy balance and glucocorticoid levels affect their infants' independence.

Another avenue of future research is to investigate the relationship between frequency of independence and growth. Previous research has found a trade‐off between infant growth and locomotor play (Malina et al. 2013; Berghänel et al. 2015). Parallel lasers could be used to measure body size in our study infants, as has been done previously with adult males in this population (Teichroeb et al. 2020). That said, an important next step is to collect more detailed behavioural data on infants to determine whether locomotor play is impacted by fruit availability, as this study only looked at whether an infant was independent or handled by an allomother. Lastly, it is important to understand whether variation in independence during early infancy has any long‐term fitness impacts. If increased independent movement during early life has any fitness benefits, it would add to our understanding of the conditions in which developmental plasticity evolved and how environmental factors affect life history trade‐offs (Stearns 1992; Nettle and Bateson 2015).

## Author Contributions

**Samantha M. Stead:** conceptualization (lead), data curation (lead), formal analysis (lead), investigation (supporting), methodology (lead), visualization (lead), writing – original draft (lead). **Edward Mujjuzi:** investigation (lead). **Julie A. Teichroeb:** funding acquisition (lead), project administration (lead), resources (lead), supervision (lead), writing – review and editing (lead).

## Data Availability

Data available in article supplementary material.
